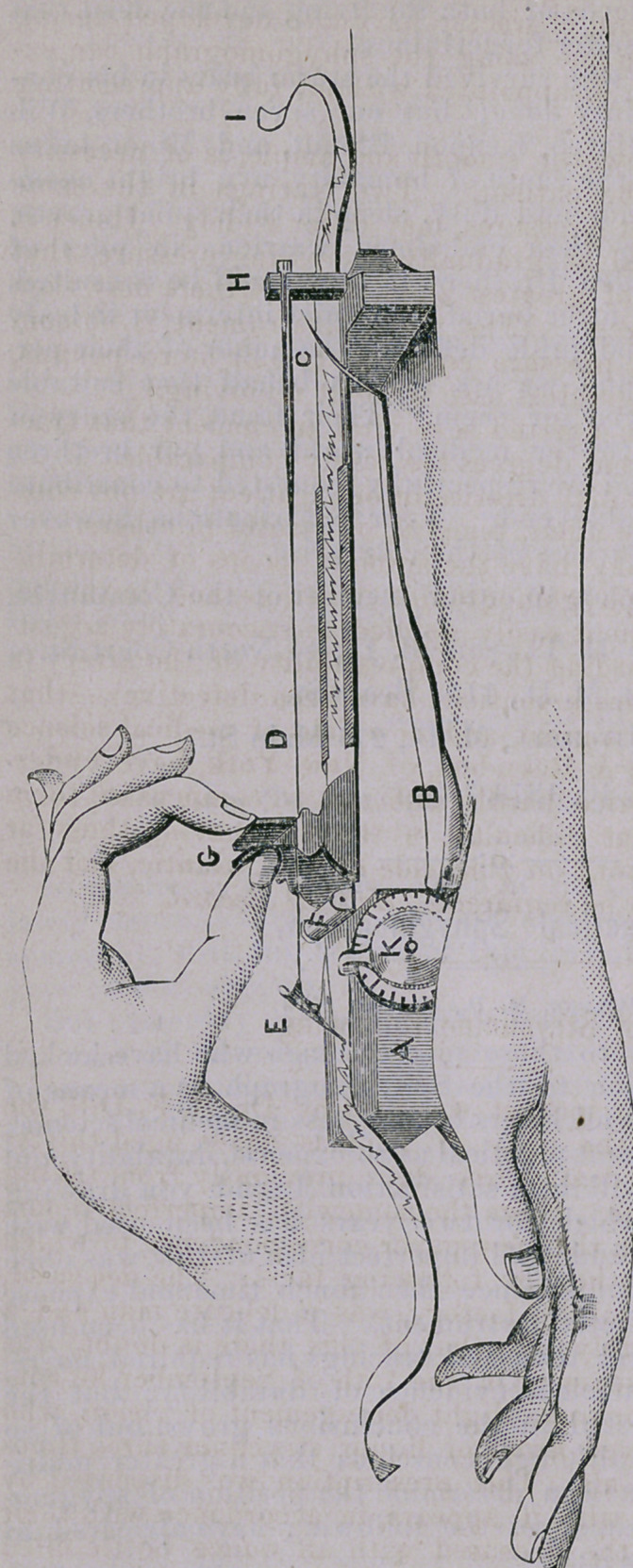# A Practical Sphygmograph

**Published:** 1874-01

**Authors:** E. Holden

**Affiliations:** Newark, N. J.


### A Practical Sphygmograph.

By E. Holden, M. D., Newark, N. J.

Sir:—Presuming that to those medical men who have looked with sanguine expectation to the Sphygmograph as a means of scientific research, and to all lovers of progress in medical science, the advent of an instrument practical as to expense, durability and facility of application will be a satisfaction. I send you drawing and description of one which for two years has been used with gratifying success. A crude and imperfect idea of this was once presented in this journal, and since then much time and expense have developed the perfected instrument. That it has been used so long, and after several thousand tracings has required no repair, will perhaps be sufficient evidence of durability; but the profession may be interested in the conclusions grown out of so many observations, and although conscious that a greater multitude of experiments alone can determine the position of spygmography as a distinct science, yet the following have appeared to me to be facts regarding it:

FIRST—That it is to be relied upon in many questions of diagnosis of obscure and simulated diseases.

SECOND.—It furnishes a means of ascertaining the condition of the arterial and venous circulation, the ability of the heart to equalize these, and the extent of impairment in disease of the heart itself.

THIRD. — It exhibits with accuracy the initiative action of remedies prior to any external and sensible manifestations of the same. Thus, in experimenting with quinine, a half-grain taken dry upon the tongue was found to show an effect upon the circulation as promptly, but in a different manner, as larger and repeated doses. The action of gelseminum and aconite was readily comparable by the tracings taken at intervals of three minutes. The opening, therefore, of a new field in experimental and therapeutical medicine is at once shown.

FOURTH.—The condition of the nervous system, in its relation to the economy as a vital power is readily exhibited, and with a dictionary of tracings, such as would result from extended experiments, the instrument may prove invaluable both in prognosis in serious disorders of the brain and spinal cord, and in the diagnosis of the numerous occult affections of these structures.

FIFTH.—Such an instrument may prove of great value in life insurance in ascertaining the eligibility of applicants.

Several facts of a general character have also developed during my investigations, and no one using the sphygomograph can expect to be otherwise than disappointed without fully appreciating them. Thus:

FIRST—No tracing, however smooth and ample, is of necessity the correct record of the patient. Two tracings in the same minute, if under different pressures, may differ widely. Hence it is essential to take several at gradually increasing pressure, that being accepted which is of greatest amplitude. Others may then be continued at that degree. (The above instrument is so constructed as to allow of a pressure equal to two or more pounds, and adjustable with the greatest ease without removing.)

SECOND—The pressure exerted is so vital an element that tracings of patients at different degrees are rarely comparable.

THIRD—Tracings of radial arteries in one patient are not comparable with those of the ulnar, femoral, or carotid in others.

That the profession may have the amplest means of determining the significance of sphygomographic writings, and in the belief that with an instrument easily applied and accurately adjustable in its means of recording the compressibility of the artery (a point in which all previous inventions have been defective),—that with such an instrument a great and new field of medical science is opened. Messrs. Otto & Reynders, of New York, have undertaken to supply it at a price hardly sufficient to compensate them for their labor; the great difficulty in the way having thus far been the cost of watchwork on this side of the Atlantic, and the limited number likely to be required.—*Medical Record.*

## Figures and Tables

**Figure f1:**